# Late-acting self-incompatibility in *Asimina triloba*: implications for the evolution of self-incompatibility in angiosperms

**DOI:** 10.1186/s12870-025-07681-6

**Published:** 2025-12-10

**Authors:** Cristina Ferrer-Blanco, Jorge Lora, Enrique López-Gómez, Aynhoa Gómez-Ollé, Noé Fernández-Pozo, José I. Hormaza

**Affiliations:** Breeding and Developmental Biology of Subtropical Fruit, Instituto de Hortofruticultura Subtropical y Mediterránea “La Mayora” (IHSM La Mayora – CSIC - UMA), Algarrobo-Costa, Málaga, 29750 Spain

**Keywords:** Asimina triloba, Early-divergent angiosperms, Evolution of reproductive barriers, Late-acting self-incompatibility, Magnoliids, Reproductive isolation, Self-incompatibility

## Abstract

**Background:**

Self-incompatibility (SI) has been reported in many angiosperm species; however, numerous SI systems remain poorly understood, particularly in early-divergent angiosperms where the molecular and genetic regulation of late-acting self-incompatibility (LSI) remains largely unknown. *Asimina triloba*, a member of the Annonaceae (order Magnoliales), provides a valuable model for investigating LSI in early-divergent flowering plants.

**Results:**

This study provides the first comprehensive investigation of LSI in *A. triloba* using macroscopic, histochemical, and transcriptomic approaches. We demonstrate that under auto-incompatible (AI) pollination, fertilization occurs but embryo development is arrested at the first zygotic division, while endosperm develops briefly, resulting in a uniseriate endosperm by 14 days after pollination (DAP). This contrasts with normal embryo development under cross-compatible (CC) pollination, where the embryo resumes division at 18–19 DAP. These postzygotic events ultimately lead to massive fruitlet abscission at 20 DAP following AI pollination. Transcriptomic analyses reveal the first differences in gene expression between early developing seeds from CC and AI pollination as early as 8 DAP, suggesting the initiation of the self-incompatibility response.

**Conclusion:**

These findings not only support the presence of a functional LSI system in *A. triloba* but also suggest the conservation of key molecular pathways across distantly related taxa. This work advances our understanding of the evolution and diversification of self-incompatibility mechanisms in early-diverging angiosperms and highlights the adaptive significance of LSI in promoting outcrossing and reproductive success.

**Supplementary Information:**

The online version contains supplementary material available at 10.1186/s12870-025-07681-6.

## Introduction

Self-incompatibility (SI) systems have evolved independently in multiple plant lineages to promote outbreeding. Since Darwin’s early observations [1], significant progress knowledge has been made in understanding the diversity of SI mechanisms in flowering plants [2–4]. Understanding the mechanisms that prevent self-fertilization in angiosperms is fundamental for unravelling the evolutionary strategies of plant reproduction. SI systems are crucial for preserving biodiversity by promoting cross-fertilization and minimize inbreeding, thereby enhancing adaptability and resilience of plant species to changing environmental conditions. In agriculture, this knowledge is instrumental to minimize SI-related challenges to improve fruit set and increase crop yields [5].

Most angiosperms are hermaphroditic, but there are different mechanisms that prevent or hinder self-fertilization. These include the spatial separation of reproductive organs (herkogamy) and temporal separation of male and female functions (dichogamy), which is often more prevalent across a wide range of plant species [6, 7]. Dichogamy can be further classified as protogynous or protandrous depending on the sequence of temporal maturation. While protandry is more common [6], protogynous dichogamy, where the stigmas become receptive before anther maturation, is nearly universal in early-divergent angiosperm clades exhibiting dichogamy [8, 9]. Besides temporal separation, many dichogamous species —typically protandrous—can also prevent self-fertilization through a SI system involving a specific molecular interaction between a male and female determinant [6, 10].

SI systems in flowering plants are typically classified morphologically into heteromorphic and homomorphic types. In heteromorphic SI, mating occurs only between individuals with different floral morphs, a condition that has independently evolved in several lineages, including the families Primulaceae, Linaceae and Turneraceae. In contrast, in homomorphic SI, compatibility is determined by the inheritance and expression of distinct *S*-phenotypes, which are determined by the *S*-locus. In gametophytic SI (GSI) the compatibility of the pollen is determined by its own haplotype, while in sporophytic SI (SSI), it is determined by the diploid genotype of the pollen producing parent. In addition to these two main systems, there is also an additional system known as late-acting SI (LSI) where the incompatibility response occurs after pollen tube growth, often in the ovary. Homomorphic GSI and SSI are the most extensively studied systems and both are controlled by a multiallelic *S*-locus. GSI is the most widely distributed SI system among angiosperms and is considered the ancestral form of SI in eudicots [11–14]. In GSI, the incompatibility reaction occurs when the *S*-allele carried by the male gametophyte corresponds to at least one of the two *S*-alleles carried by the pollen recipient. The *S*-locus contains the male-determinant *S*-haplotype-specific F-box (SFB) expressed in the pollen tube and the female-determinant *S*-RNase secreted into the style’s transmitting tissue. Pollen tube growth is blocked when *S*-haplotypes match, while non-self-pollen tubes achieve growth since non-self *S*-RNases are detoxified. In SSI, the compatibility is determined by the male diploid sporophyte (anther tapetum), which encodes the male *S*-determinant and deposited on the pollen coat during pollen development. The female *S*-protein is present in the stigma where it interacts with the male *S*-determinant [12, 15].

Although GSI and SSI are the most widely reported SI mechanisms in angiosperms, their distribution is still limited across the 415 recognized families of flowering plants [16]. A recent review [17] analyzing 5686 hermaphroditic taxa shows that 55% of them are self-compatible and the rest show some degree of self-sterility with 20% of the families showing total (20%) or partial (8%) SI. To our knowledge, GSI has been conclusively identified in species from 18 families and SSI in species from 6 families [12]. Considering that SI has been reported in approximately 100 families of flowering plants, and that its occurrence may have been underestimated [18], many SI systems remain unexplored, especially in terms of their molecular and genetic regulation. Most molecular and genetic insights into SI have come from a few model families: GSI in Solanaceae, Rosaceae, and Plantaginaceae and SSI in Brassicaceae and Papaveraceae [12].

Among these less explored SI systems, is LSI, also known as ovarian incompatibility or pistillate sorting [19], a system that has been scarcely analysed compared to GSI or SSI. In LSI, self-pollen tubes reach the ovary, often even reaching the ovule and achieving fertilization, yet the plant remains self-sterile [12]. LSI can be either prezygotic or postzygotic, depending on whether fertilization occurs. Postzygotic LSI may be confused with inbreeding depression (ID), although embryo abortion in ID is typically more variable than in postzygotic LSI [20]. Although the genetic basis of LSI remains poorly understood, it is hypothesized to involve gametophytic control [19]. This is supported in crossbreeding experiments in species like *Acacia retinoides* [21], while *Theobroma cacao* shows a more complex system involving both gametophytic and sporophytic control via three independent loci [22, 23]. In *Gasteria* spp., two or more loci are thought to control LSI [24], and a single locus is suggested to control postzygotic LSI in *Asclepias exaltata* [20].

Few studies have explored the genetic regulation of LSI beyond crossbreeding experiments. In *Xanthoceras sorbifolium*, transcriptomic analysis of postzygotic LSI revealed 274 genes specifically expressed in self-pollinated seeds [25]. In *Theobroma cacao*, six classes of candidate genes have been suggested to govern LSI, including calcium/calmodulin-dependent kinases, serine/threonine phosphatases, serine/threonine kinase, “Plant self-incompatibility S1,” FAR1 protein, and locus-*S* glycoprotein, in addition to a gene associated with pollen rupture [26].

Understanding SI in early-divergent angiosperms is critical for reconstructing the evolutionary history of plant reproductive strategies. However, while self-sterility attributed to an SI system has been reported in a few early-divergent angiosperms, there have been no studies on its molecular and genetic regulation. This is the case for stigmatic SI reported in *Trimenia moorei* (Trimeniaceae) [27] and *Saururus cernuus* (Saururaceae) [28], as well as for LSI reported in *Pseudowintera colorata* and *P. axillaris* (Winteraceae) [29, 30]. Self-sterility has also been reported in other early-divergent angiosperms, but without histological evidence or a genetic basis associated with a SI system. For example, three species of *Magnolia* (Magnoliaceae) have been reported as SI, although the type of SI is unclear [31]. In *Illicium floridanum*, GSI was initially reported [32], but later studies showed that self-pollen can grow to reach the ovule, suggesting that it should be considered as LSI [33], although more recent studies have attributed the observed patterns to inbreeding depression [34].

In the Annonaceae family, within the ancient eumagnolid lineage, SI has also been reported in *Asimina triloba*, primarily based on observations of fruit production in the field [35, 36]. It has been suggested that *A. parviflora*, which appears to have a similar breeding system to *A. triloba*, exhibits some signs of postzygotic incompatibility or inbreeding depression, as indicated by lower fruit and seed set [37]. The Annonaceae family comprises 110 genera and approximately 2,450 species of trees, shrubs, and lianas [38], many of which produce edible fruits in tropical and subtropical regions. While nearly all species within the Annonaceae originate from tropical and subtropical climates, the genus *Asimina* is unique as the only group in the family with species adapted to cold climates. *A. trilobal*, the American pawpaw, is an emerging fruit crop that is almost exclusively produced in North America, but it has a clear potential for expansion into other regions with temperate climates [39, 40].

The self-sterility observed in *A. triloba*, although almost unexplored, is particularly interesting because it represents a unique feature in the otherwise self-compatible species of the Annonaceae. Given the phylogenetic position of the Annonaceae, understanding the mechanisms of SI in this group is crucial, not only for gaining deeper insights into self-incompatibility itself but also for understanding the evolutionary history of incompatibility systems in flowering plants. To address this knowledge gap, this study aims to investigate whether *A. triloba* exhibits a late-acting self-incompatibility system, and to elucidate the molecular and genetic mechanisms underlying this phenomenon. To achieve this, a comparative study of early seed development is conducted, examining both auto-incompatible (AI) and cross-compatible (CC) pollination through histochemical and transcriptomic analyses.

## Materials and methods

### Plant material

The flowers of *A. triloba* have a conical arrangement of the gynoecium and androecium and exhibit protogynous dichogamy with an extended stigmatic receptive period that may facilitate geitonogamy [41]. For this study, six self-incompatible adult trees of *Asimina triloba* [L.] Dunal grown from seed were utilized. The trees were located at two locations: three at IHSM La Mayora UMA-CSIC (Málaga, Spain), during the flowering seasons from 2019 to 2022 (April-May), and three at the Arnold Arboretum of Harvard University (Boston, EEUU), during May 2022. All six trees were genetically distinct, making them cross-compatible with each other but self-incompatible.

### Pollination procedures

Flowers at anthesis were enclosed in white nylon mesh bags to prevent contamination by external pollen and allow controlled cross-compatible (CC) and auto-incompatible (AI) hand pollinations. Bagged flowers were examined twice daily to determine the period of maximum stigmatic receptivity, indicated by abundant secretion rich in cell-wall-related arabinogalactan proteins (AGPs) and insoluble polysaccharides, which facilitate pollen adhesion, germination, and pollen tube growth [41]. For hand pollination (both AI and CC pollination), the nylon bag was removed, the female state of the flower was confirmed, and the stigmas were inspected under a magnifying glass to ensure absence of any pollen. Pollen was then applied with a brush until the entire stigma surface was covered. Finally, the flower was re-bagged with the nylon bag for 48 h before final removal.

### Fruit development

To determine the timing of fruit abscission following CC and AI pollinations and to compare fruit development between both pollination types, three immature fruits from each pollination type were sequentially collected at six developmental stages (*n* = 36; 6 stages x 2 pollination types x 3 fruits), starting 14 days after pollination—when fruit dimensions were statistically similar. The fruits were then weighed using a precision scale, and their length and width were measured with a digital caliper. Duncan’s multiple range test (*P* ≤ 0.05) was used to separate means, and all statistical analyses were conducted using SPSS version 12.0.

### Growth of compatible and incompatible pollen tubes

To study potential differences in pollen germination and tube growth between CC and AI pollinations, flowers were collected at their peak stigmatic receptivity and placed in a high-performance growth chamber (average temperature: 16 °C, relative humidity: 80%) to minimize potential environmental effects. On the tree, the flowers were previously bagged to prevent the arrival of unwanted pollen. In the growth chamber, CC and AI pollinations were performed simultaneously. Because we previously observed that the first pollen tube reaches the locule approximately 3 h after germination [41], pistils were collected 9 h after pollination. These pistils were then squashed and stained with 0.1% aniline blue in 0.1 M PO_4_K_3_ [42, 43]. Stained samples were visualized under a Leica DM2500 epifluorescence microscope with 515–560 nm and LP 590 filters. The total number of ovules per pistil (4,5 ± 1,4; *n* = 56 flowers, [44]) and the number of pollinated ovules were counted, considering an ovule as fertilized if it showed a pollen tube at the micropyle [45]. Mean equality was assessed through ANOVA, followed by a Duncan test (*P* < 0.01) using Rstudio software. Images were captured with a Leica camera attached to the microscope connected to a computer, using LAS v4.5 software.

### Histochemical preparations

For the comparative study of ovule and embryo development, flowers were sequentially collected at 1, 2, 3, 4, 13, 14, 18, 19 and 20 days after pollination, and fixed in either 4% paraformaldehyde in phosphate-buffered saline (PBS) at pH 7.3 (samples from IHSM La Mayora) or 1% acrolein (Polysciences, New Orleans, Louisiana, USA) in 1X PIPES buffer (50 mmol/L PIPES, 1 mmol/L MgSO_4_, 5 mmol/L EGTA, pH 6.8) (samples from the Arnold Arboretum at Harvard University) for 24 h at room temperature. The samples were then dehydrated in a graded ethanol series, embedded in Technovit 8100 (Kulzer & Co, Wehrheim, Germany) for material fixed with 4% paraformaldehyde, or in JB-4 glycol methacrylate (Electron Microscopy Sciences, Hatfield, PA, USA) for material fixed with 1% acrolein, and sectioned at 2–4 μm. The sections were stained with periodic acid-Schiff (PAS) for insoluble carbohydrates, and with toluidine blue (Sigma-Aldrich) for general histological observations [46].

### RNA extraction from early developing seeds

For each pollination type and time point, three flowers were collected: three CC- or AI-pollinated flowers at 4 days after pollination (DAP), another set at 8 DAP, and a third at 15 DAP, resulting in a total of 18 biological replicates. The pistils of each flower were dissected to obtain the developing seeds, which were placed in Eppendorf tubes with cetyltrimethylammonium bromide (CTAB) buffer. Immediately afterward, RNA extraction from these fresh early seeds was performed using a specific protocol that combines CTAB [47] and the RNeasy Plant Mini Kit (Qiagen, Stanford, CA), following the manufacturer’s instructions.

### Sequencing and analysis

Library preparation (poly-A enrichment) and mRNA (cDNA) sequencing were conducted at the central sequencing facilities of Novogene Company Limited (Cambridge, United Kingdom) using an Illumina NovaSeq PE150 sequencer, aiming to obtain 30 million read pairs per sample. Raw reads were assessed with FastQC v.0.11.9 (https://www.bioinformatics.babraham.ac.uk/projects/fastqc) and MultiQC v.1.13a [48]. Quality filtering and adapter trimming were performed using Trimmomatic v.0.39 [49], with the parameters ILLUMINACLIP: TruSeq3-PE.fa:2:30:10 LEADING:3 TRAILING:3 SLIDINGWINDOW:4:15 MINLEN:36. The processed reads were mapped to the *Asimina triloba* v105 genome (unpublished) using HISAT2 v.2.2.1 [50] and subsequently converted to sorted BAM format using SAMtools v.1.16 [51]. Gene expression was quantified at the exon level using FeatureCounts from the Subread package v.2.0.3 [52].

Principal component analysis (PCA), specific gene identification, and functional enrichment analyses were conducted using R v.4.4.1. Genes were normalized to Counts per Million (CPM) with EdgeR package [53], and PCA clustering was performed using the prcomp function from stats package. Raw gene counts were normalized to Transcripts per Million (TPM) using the convertCounts function from the R package DGEobj.utils, and genes with a minimum expression threshold of TPM ≥ 1 in control conditions were considered for specific gene identification. DESeq2 v1.46.0 [54] was employed for normalizing gene expression data and identifying differentially expressed genes (DEGs). The orthologs between *Asimina triloba* and *Arabidopsis thaliana* were identified using Diamond BLASTp v2.1.8.162 [55]. Best hits of the comparison between *A. triloba* and *A. thaliana* and vice versa were retained, and matches with a bit-score below 45 were filtered out. The proteins of *Arabidopsis thaliana* most similar to the DEGs found were utilized for the functional enrichment analysis, which was performed using a custom script (https://github.com/bullones/FunRichR) based on the clusterProfiler package v.4.14.6 [56].

For homologs associated with embryo development in *Arabidopsis thaliana* and potential LSI candidate genes from *Theobroma cacao* (Table S1), differential expression data normalized to TPM were evaluated using ANOVA. Although both species are phylogenetically distant from *A. triloba*, they were selected because of the extensive information available in those two species. Duncan’s multiple range test was applied for mean separation (*P* ≤ 0.05). Statistical analyses were performed using SPSS 12.0.

## Results

### *A. triloba *exhibits a post-zygotic LSI

**Fig. 1:**
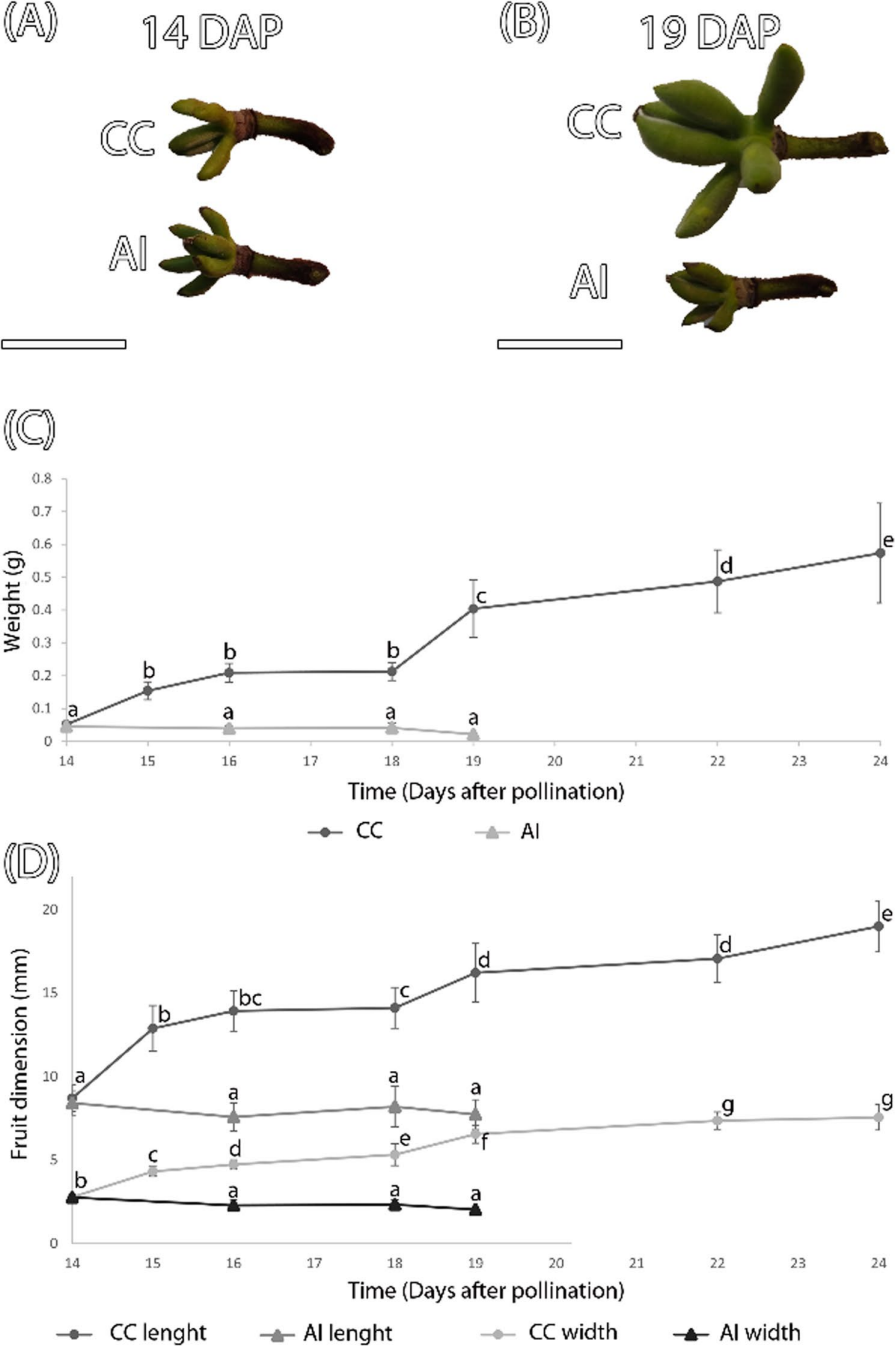
Early fruit development in cross-compatible (CC) and auto-incompatible (AI) pollinations. **A-B** Fruits at 14 (A) and 19 (B) days after pollination (DAP) from CC and AI pollinations. **C-D** Mean pistil weight (C) and fruit dimension (D) after anthesis in CC and AI pollinations, with error bars representing standard deviation (s.d.). Different letters within each trait (weight, length, and width) indicate significant differences (*P* ≤ 0.05; Duncan’s multiple range test). Letters are not comparable across traits. Bars = 2 cm

To assess the occurrence of an incompatibility reaction, we conducted a comparative study of fruit development by measuring the weight, length, and thickness of fruits resulting from cross-compatible (CC) and auto-incompatible (AI) pollinations. At 14 days after pollination (DAP), no significant differences were observed in fruit weight and dimension between both types of crosses (mean weight *P*-value 0.13, mean width *P*-value 0.66, and mean length *P*-value 0.17, α = 0.05). However, in subsequent days, the immature fruits from CC pollinations showed an increasing variability, whereas those from AI pollinations did not, ultimately leading to the abscission of the fruits resulting from AI pollinations by 20 DAP (*n* = 26 fruits). Meanwhile, unpollinated flowers abscised four days after anther dehiscence (*n* = 10 fruits) (Fig. 1).

To determine which type of SI is present in *A. triloba*, we first analyzed pollen tube growth in both CC and AI pollinations. Consistent with previous reports [41], pollen tubes from CC pollinations reached the ovary 3 h after pollination. In this study, we evaluated pollen tube arrival at the micropyle 9 h after pollination in both CC and AI pollinations. We quantified the proportion of ovules showing pollen tube at the micropyle, finding no significant differences between pollination types: 9% ± 8% (*n* = 183) ovules for CC and 6% ± 5% (*n* = 201) ovules for AI. These values represent the mean percentage ± standard deviation of ovules per carpel that showed pollen tubes at the micropyle, based on fluorescence microscopy observations. While the presence of a pollen tube at the micropyle does not guarantee fertilization, these results demonstrate that pollen tubes from AI pollinations successfully traverse the stigma, style, and ovary. This supports the hypothesis that SI in *Asimina triloba* is not due to prezygotic pollen tube arrest, and is consistent with a LSI mechanism.

**Fig. 2:**
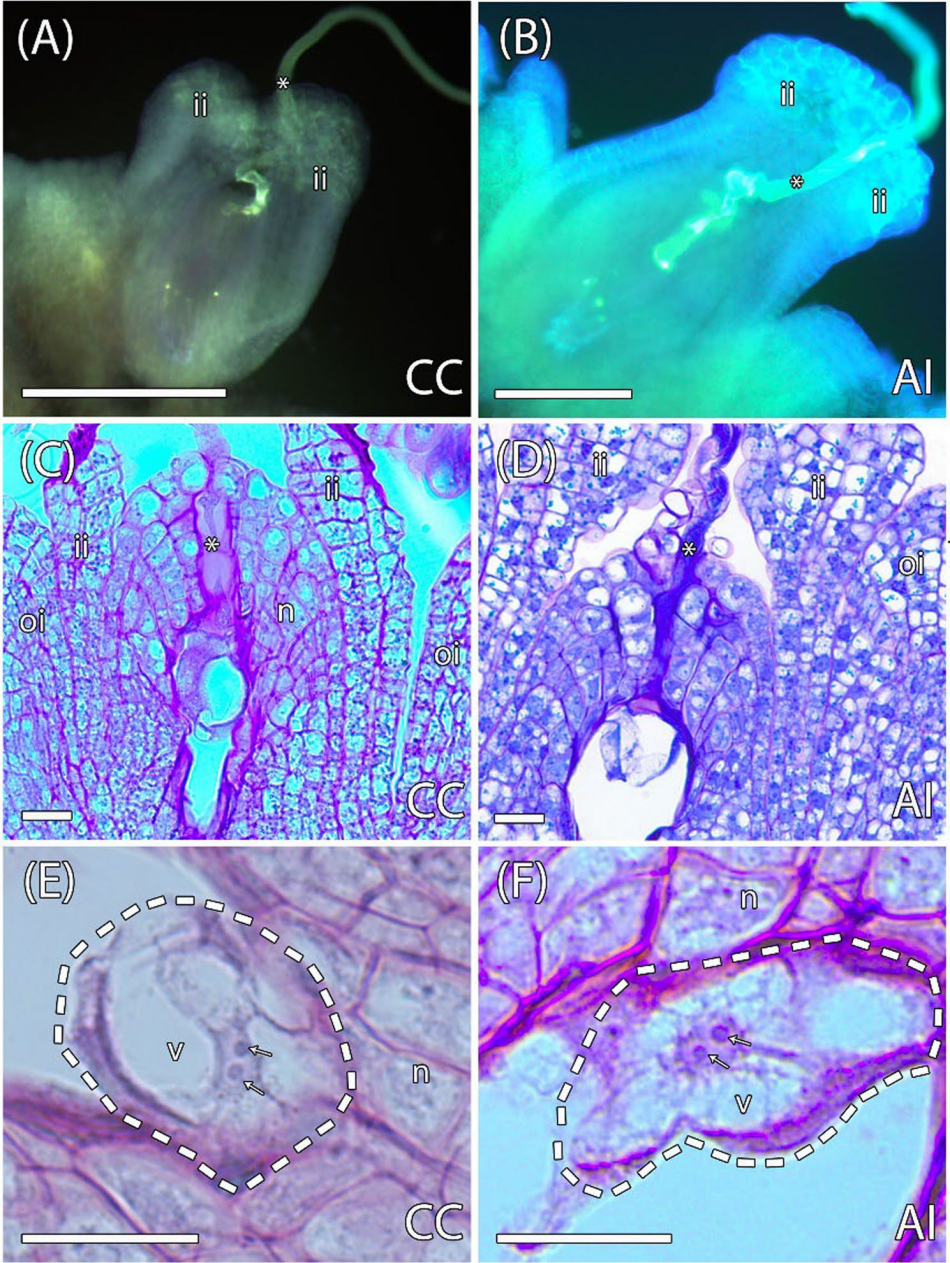
Pollen tube growth and fertilization in cross-compatible (CC) and auto-incompatible (AI) pollinations. **A-B** Pollen tubes (asteriks) in the micropyle from CC (A) and AI pollinations (B) visualized with aniline blue. **C-D** Longitudinal sections of the pistil from CC (C) and AI pollinations (D), showing pollen tubes (asterisks) in the micropyle reaching the egg apparatus. **E-F **Zygotes (outlined by dashed lines) from CC (E) and AI pollinations (F) displaying two prominent nucleoli (arrows) and dense, periodic acid–Schiff (PAS)-reagent-positive cytoplasmic loops. ii, inner integument; n, nucellus; oi, outer integument; v, vacuole. Bars: (A-B) 100 μm; (C-F) 20 μm

To further investigate the timing of the incompatibility response, we performed a histological analysis to track fertilization events. Semi-thin sections revealed pollen tube penetration through the nucellus at the micropylar end in ovules of both CC and AI pollinated flowers. This process was similar in both cases, forming a cytoplasmic loop over the egg apparatus at 1–2 DAP (Fig. 2A-D). By 3–4 DAP, the presence of a zygote with two nucleoli and large amorphous vacuoles was observed in ovules of both pollinations, representing 34.1% of the analyzed ovules in CC (*n* = 176 from 4 flowers) and 31% in AI (*n* = 106 from 4 flowers) (Fig. 2E-F), as previously reported in *A. triloba* [44], confirming that fertilization had occurred even following AI pollination. Percentages of pollen tube presence at the micropyle (9% in CC and 6% in AI) and zygote formation (34.1% and 31%) are not directly comparable, as they refer to different sampling bases and time points (per carpel at 9 h vs. total ovules at 3–4 DAP).

### At the cellular-level, *A. triloba* shows delayed seed development following AI pollination

**Fig. 3:**
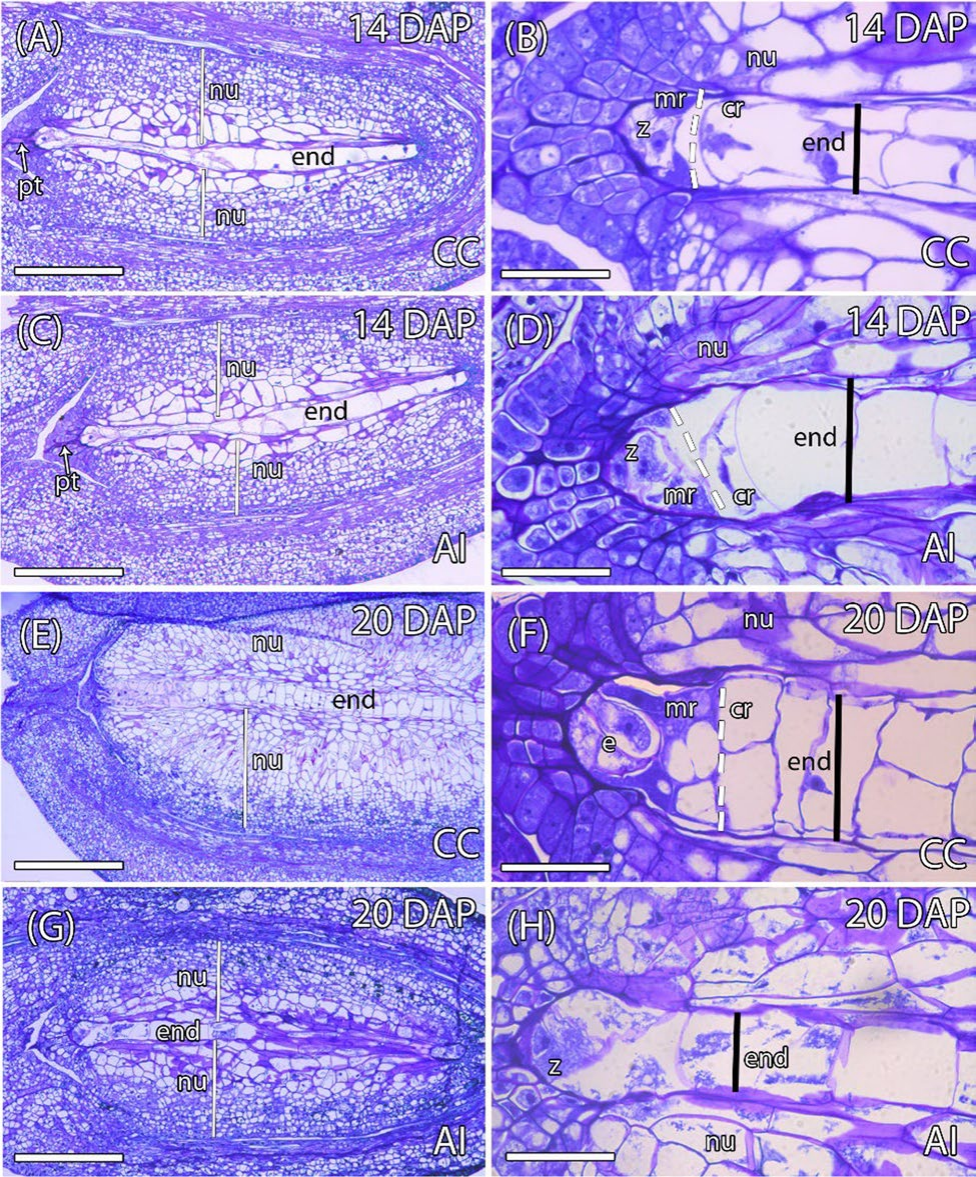
Longitudinal sections of early developing seeds from cross-compatible (CC) and auto-incompatible (AI) pollinations. **A** Early seed of a CC pollination at 14 days after pollination (DAP) with uniseriate endosperm. **B** Detail of the micropylar region of the endosperm in a CC pollination at 14 DAP, showing a zygote. **C** Early developing seed of an AI pollination at 14 DAP with uniseriate endosperm. **D** Detail of the micropylar region of the endosperm in an AI pollination at 14 DAP, showing a zygote. **E** Early developing seed of a CC pollination at 20 DAP with multiseriate endosperm. **F** Detail of the micropylar region of the endosperm in a CC pollination at 20 DAP, showing a embryo. **G** Degenerated seed of an AI pollination at 20 DAP. **H** Detail of the degenerated endosperm of an AI pollination at 20 DAP. cr. chalazal region; e, embryo; end, endosperm; mr, micropylar region; nu, nucellus; pt, pollen tube; z, zygote. Bars = 20 μm

Fruitlets resulting from AI pollinations consistently abscised by 20 DAP (Fig. 1). To evaluate the cellular-level differences between CC and AI pollinations, we conducted a histological study of early embryo and endosperm development in both crosses. Initially, both pollination types showed similar patterns of seed development. There was an increase in the size of the nucellus, mainly due to increased cell division and the enlargement of cells in contact with the developing endosperm, which also exhibited a prominent large vacuole (Fig. 3A-B). Endosperm differentiation was also comparable between CC and AI pollinations, characterized by a well-defined micropylar region with pyramidal-shaped cells containing dense PAS-stained cytoplasm, and a chalazal region with larger, vacuolated cells (Fig. 3B, D). In AI pollinations, the zygote was observed after 13–14 DAP, although it very rarely showed its first division, which typically occurs at 18–19 DAP in CC pollinations. Although no zygote development was observed in AI pollinations at 13–14 DAP, endosperm development was clearly evident, forming a uniseriate central row composed of about 8–15 large, vacuolated cells (Fig. 3 C, D). At this stage of development, remnants of the pollen tube were commonly observed in immature seeds from AI pollinations (Fig. 3 C). These results suggest that until approximately 13–14 DAP, the development of the endosperm and zygote was similar between CC and AI pollinations (Fig. 3 nullA-D).

However, by 18–19 DAP, developmental differences between CC and AI pollinations became apparent (Fig. 3E-H). After CC pollinations, both the embryo and endosperm continued to develop normally, whereas development in AI pollinations was arrested. By 20 DAP, in CC pollinated plants, the micropylar domain was still observed, but this was not the case in AI pollinated plants, which exhibited clear signs of tissue degeneration, remaining in a developmental state similar to that observed at 13–14 DAP (Fig. 3E–H).

### Gene expression in early developing seeds from CC and AI pollinations

**Fig. 4:**
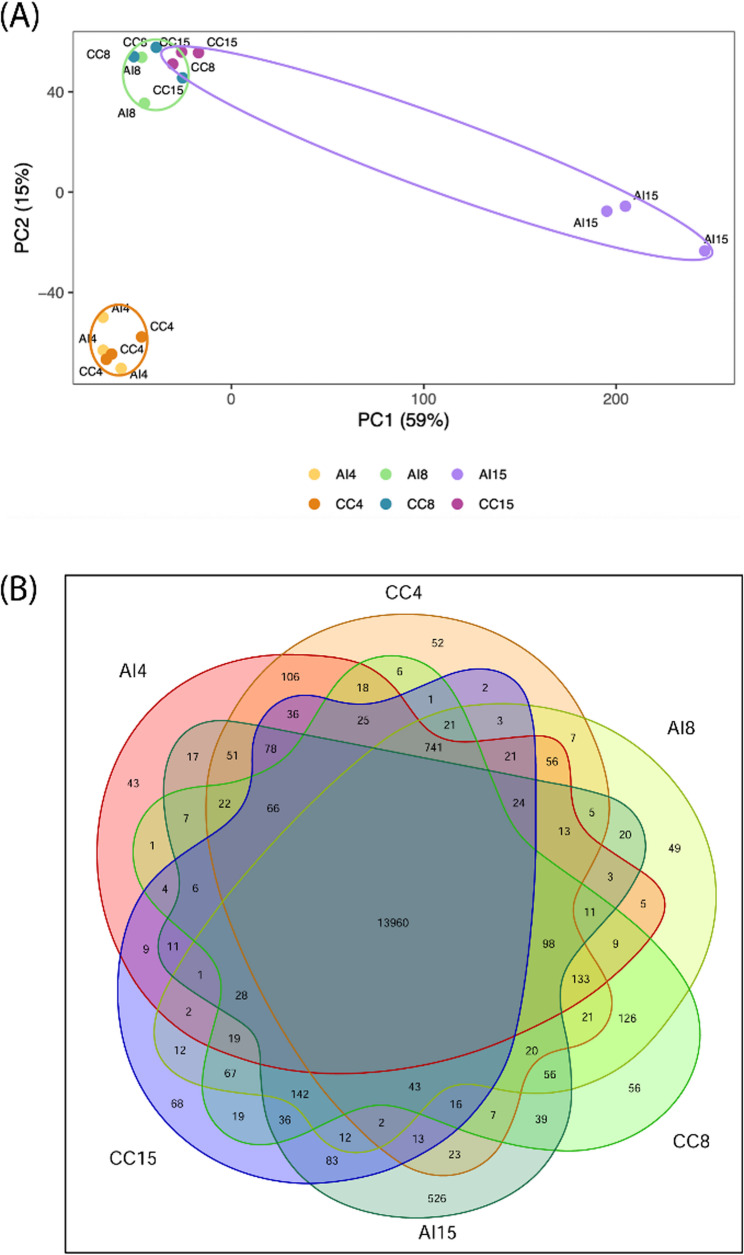
AI samples at 15 DAP show greater differences with the rest of the samples and present a greater number of specifically expressed genes. **A** Principal Component Analysis (PCA) of gene expression across all samples. **B** Venn diagram showing shared and specific transcripts across all samples

To gain insight into the observed differences at the cellular level between both types of crosses, we evaluated the gene expression at 4, 8, and 15 DAP through a transcriptome study. A principal component analysis (PCA) of gene expression across all samples showed a clear clustering pattern where, at 4 DAP, the CC and AI samples grouped together (Fig. 4, in orange), indicating similar transcriptional profiles at this early stage. A similar pattern was observed at 8 DAP, where both pollination types again grouped together (Fig. 4, in green), suggesting that early transcriptional programs remained largely consistent between CC and AI seeds through this stage. However, by 15 DAP, distinct differences emerged. The CC samples at 15 DAP clustered closely with those at 8 DAP, indicating continued progression of developmental gene expression programs. In contrast, AI samples at 15 DAP shown to be more distant to all other samples, especially in the first component, which is responsible of 59% of the differences (X axis). This observation was further supported by a correlation analysis (Fig. S1) and a specific gene expression analysis (Fig. 4B), where the AI sample at 15 DAP showed much lower correlation to all other samples, and the highest number of specific genes.

To identify the specific genes uniquely expressed in each condition, transcripts with a minimum abundance of one transcript per million (TPM) were analyzed. At 4 DAP, 52 transcripts were specifically expressed in CC pollination and 43 in AI pollination. A similar pattern was observed at 8 DAP, with 56 (CC) and 49 (AI) specifically expressed transcripts. However, at 15 DAP, while CC pollination maintained a similar number of specifically expressed transcripts (68), AI pollination showed a marked increase, with 526 uniquely expressed transcripts—approximately a tenfold rise compared to all other samples (Fig. 4B). To understand the differences of the AI sample at 15 DAP to all other samples, a gene function enrichment based on the GO Biological Processes of the specific genes in this sample was performed, revealing terms associated to *plant organ senescence*, *secondary metabolism biosynthesis*, *response to salicylic acid* or *response to hypox*i.a. among others, suggesting the activation of processes potentially involved in fruit abscission (Fig. S2).

In addition, Differentially Expressed Genes (DEG) between CC and AI samples at each timepoint were identified: At 4 DAP, no significant DEGs were found, suggesting that the incompatibility reaction had not yet taken place. By 8 DAP, 476 DEGs were identified with 354 upregulated in CC (CC > AI) and 122 downregulated (CC < AI), indicating the initiation of the incompatibility response, with threefold upregulated genes in the compatible cross (Table S2). At 15 DAP, 13,397 DEGs were observed with 6607 upregulated in CC (CC > AI) and 6790 downregulated (CC < AI), showing huge differences of expression between the AI and the CC samples (Fig. S3). These findings align with the differences observed in the PCA, correlation and specific genes between AI at 15 DAP and all the other samples at all timepoints. These results indicate a large number of differentially expressed genes, suggesting that the molecular mechanism of the self-incompatibility system is initiated before 15 DAP, although histochemically, differences were not yet noticeable.

**Fig. 5:**
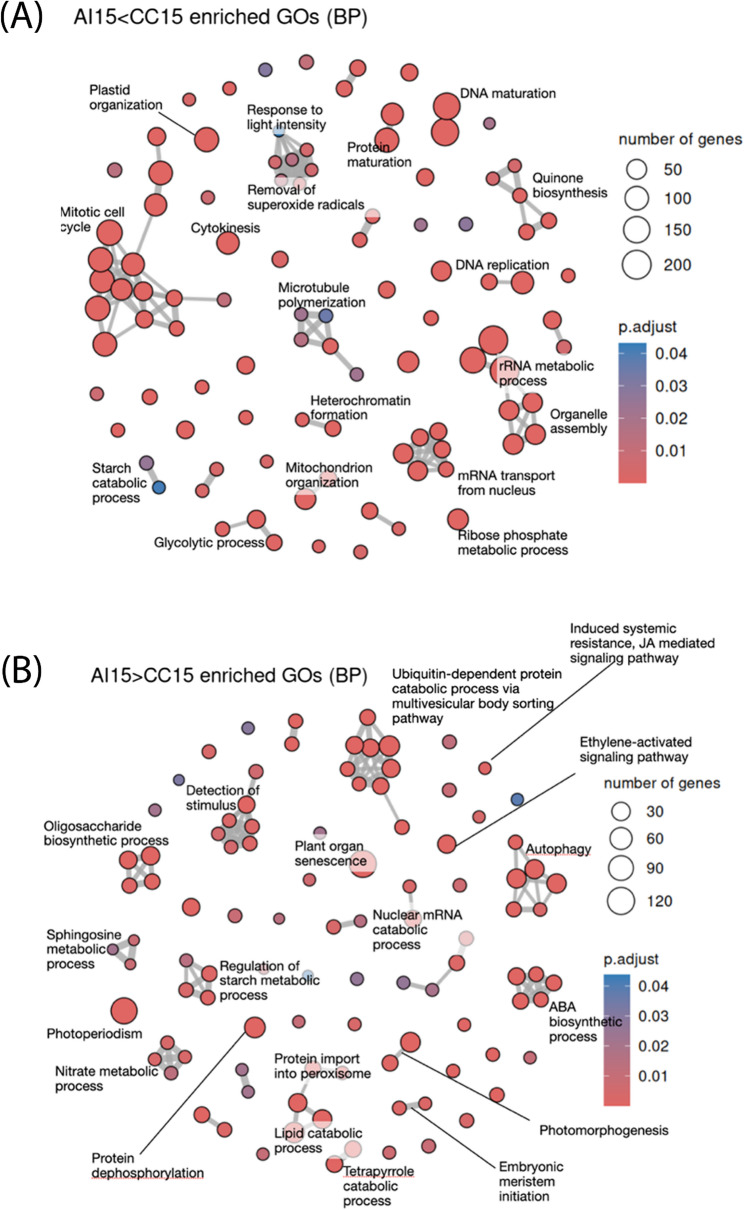
Network of the biological processes associated with differentially expressed genes (DEG) between cross-compatible (CC) and auto-incompatible (AI) pollination at 15 days after pollination (DAP). **A** Enriched processes in upregulated genes in CC pollination compared to AI pollination. **B** Enriched processes in upregulated genes in AI pollination compared to CC pollination

Due to the huge number of differences between CC and AI at 15 DAP it is complex to discern among 13 thousand genes which ones are responsible of the incompatibility system. The results at this time seem to show the differences between fruits that continue developing in CC, showing a gene expression similar to the expression data at 8 DAP, and fruits that will be discarded and show the expression of multiple genes related to stress to induce the abscission of the fruit in AI at 15 DAP. In CC, enriched biological processes include cell division, DNA replication, organelle organization, and metabolism (Fig. 5A). In contrast, AI shows enrichment in senescence, stress signalling (ethylene, JA, ABA), catabolic processes, and developmental arrest (Fig. 5B).

To get more insights about the processes occurring during AI pollination at 15 DAP, a gene set enrichment analysis of the DEGs between AI samples at 8 DAP and 15 DAP was performed, showing similar results to those observed between CC and AI at 15 DAP (Fig. S4).

**Fig. 6:**
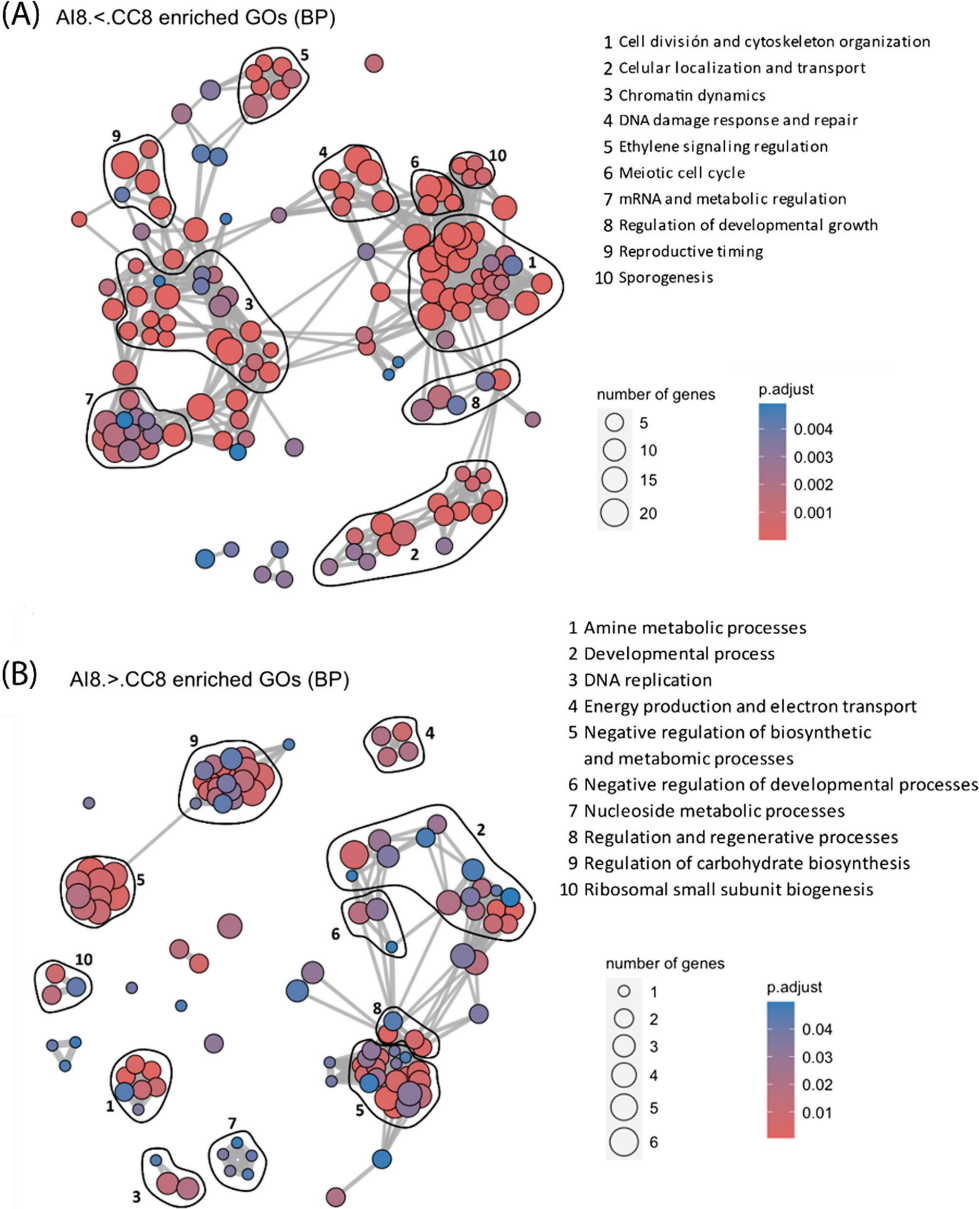
Network of the biological processes associated with differentially expressed genes (DEGs) between cross-compatible (CC) and auto-incompatible (AI) pollination at 8 days after pollination (DAP). **A** Enriched processes in upregulated genes in CC pollination compared to AI pollination. **B** Enriched processes in upregulated genes in AI pollination compared to CC pollination

At the transcriptomic level, 8 DAP appears to be closer to the initiation of the self-incompatibility response, since there were almost no DEGs at 4 DAP and gene expression at 15 DAP was already very different. To understand the biological significance of the 476 DEGs identified between CC and AI pollinations at 8 DAP (Table S2), a Gene Ontology enrichment analysis was performed. Genes up-regulated in CC pollination were primarily associated with biological processes related to cell division, cytoskeleton organization, cellular localization, growth, epigenetic regulation and transport, highlighting the terms embryo sac development and vegetative to reproductive phase transition of meristem (Fig. 6A). In contrast, genes up-regulated in AI pollination contained a lower number of genes involved in enriched biological processes (note scale in Fig. 6), and were mainly associated with the biological processes of regulation of signaling, regulation of development, and negative regulation of transcription. Taken together, at 8 DAP, the GO enrichment analysis revealed that both crosses clearly engage different biological processes. CC pollination was more directed towards proliferation and developmental processes in comparison with the AI pollination early seeds (Fig. 6).

Interestingly, among the DEG at 8 DAP, 11 were associated with *EMBRYO-DEFECTIVE* mutants, of which 9 were overexpressed in CC pollination (Table S2). The significant upregulation of these *EMBRYO-DEFECTIVE*-related genes in CC pollinations suggests a potential regulatory role in facilitating successful embryo development in CC scenarios.

Beyond general transcriptomic differences, we specifically analyzed genes involved in embryo development, focusing on those related to hybrid incompatibility, fertilization, early embryogenesis regulation, endosperm development, and *ARGONAUTE* (*AGO*) genes in *Arabidopsis*, as well as putative candidates for LSI identified in *Theobroma cacao* (Fig. S5, Table S1).

For these genes, the most significant differences were observed at 15 DAP, where a higher number of DEGs were exhibited between CC and AI pollinations, reflecting distinct biological functions.

At 4 DAP, an homolog of *EGG CELL* was upregulated in CC compared to AI pollination. Conversely, a notable downregulation of *Zygote Stage Zeus 1* (*ZEU1*) homologs was observed in CC compared to AI pollination. Interestingly, a lectin receptor-like kinase (LecRLK) from the *S*-locus (AT4G21390) was also downregulated in CC compared to AI pollination at 4 DAP, and remained consistently downregulated at both 8 and 15 DAP.

Regarding LSI candidates in CC crosses, we identified the upregulation of homologs of *FAR1*-related sequence *FRS1/2*, *FRS3/5* and *FRS9* at 4 DAP and *FRS5* and *FRS9* at 8 DAP. At 8 DAP the homolog of *BAM3* was also upregulated at 8 DAP. At 15 DAP, homologs of *BAM1/2*, *BAM3*, and one serine/threonine protein kinase were upregulated. Conversely, at 15 DAP, homologs of a voltage-dependent L-type calcium channel subunit (Tc04_g000160), U-box domain-containing protein 40 *ARC1* (Tc04_g000330), *CONVERGENCE OF BLUE LIGHT AND CO2 2* (*CBC2*) (Tc04_g000810), nine *FAR1-related sequences* and two serine/threonine protein kinase were downregulated (Table S1, Fig. S5).

All the gene expression data have been included in the expression atlas hosted at IHSM Subtropicals (https://www.subtropihsmcsic.uma.es/easy_gdb/index.php). The provisional name of each gene has been extracted from the *A. triloba* draft genome v105 and will be updated in the expression atlas after publication of the genome.

## Discussion

In this study, we observed LSI in the early-diverging angiosperm *A. triloba*. In AI pollinations, at the macroscopic level, massive drop-off of immature fruits was observed at 20 DAP. At the cellular level, differences between AI and CC pollinations were evident around 13–14 DAP, while at the transcriptomic level, differences were detectable at 8 DAP, with limited early differences at 4 DAP.

### *Asimina triloba* shows a dual approach to prevent self-fertilization

*Asimina triloba* exhibits protogynous dichogamy, a reproductive strategy characterized by the temporal separation between stigmatic receptivity and anther dehiscence, which serves to hinder self-pollination. This mechanism is common among early-diverging angiosperms and could be considered the ancestral condition in flowering plants [8, 10]. The effectiveness of this temporal separation in preventing self-fertilization largely depends on the duration of stigmatic receptivity, which typically lasts 1 or 2 days in most species [57]. However, this effectiveness has been scarcely evaluated in the Annonaceae. Although dichogamy seems to be effective in preventing self-fertilization, some selfed fruits can still occur in some cases, such in *Annona cherimola*, from the overlap between stigmatic receptivity and anther dehiscence. Environmental conditions can influence the length of stigmatic receptivity by either promoting self-fertilization or increasing the temporal separation between the female and male phases. This plasticity may act as a bet-hedging strategy, promoting outcrossing while at the same time ensuring reproductive success [58]. *A. triloba*, however, is a special case within this dichogamous system since the duration of stigmatic receptivity is particularly long, lasting up to 11 days, which is uncommon in species that exhibit protogynous dichogamy [41]. The efficiency of the temporal separation between the female and male phases has not been evaluated in *A. triloba*, but given the long duration of stigmatic receptivity, this effectiveness could be compromised, and it is possible that the low efficiency may have promoted a second mechanism to avoid self-crossing, in this case, a postzygotic SI.

The presence of an incompatibility mechanism is very rare in Annonaceae [59, 60], and the occurrence of protogynous dichogamy along with an incompatibility system, as seen in *A. triloba*, is an extremely rare trait in flowering plants. Although many dichogamous species exhibit SI, protogyny has been correlated with self-compatibility, while protandry is associated with SI [10]. Among members of the ANITA grade (Amborella, Nymphaeales, Illiciales (Illicium and related genera), Trimeniaceae, and Austrobaileyales), SI has been suggested or confirmed in a few cases such some species of the genera *Nuphar* [61], and *Nymphaea* [62] in the Nymphaeaceae (Nymphaeales) and it has also been proposed but not fully understood in *Austrobaileya scandens* [63, 64] (Austrobaileyales). Among other early-divergent angiosperms, the incompatibility mechanism is established as stigmatic SI in *Trimenia moorei* and *Saururus cernuus* [27, 28], LSI in *Pseudowintera colorata* and *P. axillaris* [29, 30], while the mechanism remains unclear in *Magnolia* (Magnoliaceae) [31] and was reclassified as inbreeding depression in *Illicium floridanum* [34].

Thus, the unexplored SI mechanisms in early-divergent angiosperms contrast with the well-studied molecular and genetic regulation of GSI and SSI systems in monocots and eudicots, while LSI remains particularly unknown in these groups, with only a few studies available.

### LSI occurs after fertilization

LSI has been primarily studied through studies of fruit set and seed production following self-pollination [59, 65–67], or in more detail, through the visualization of pollen tubes penetrating the micropyle [68, 69]. Inbreeding depression has been linked to many species with LSI, often considered the more likely explanation for low fruit set following self-pollination. However, it has been suggested that LSI is characterized by uniform ovule rejection at the same developmental stage across individuals, whereas inbreeding depression (ID) involves variable rejection stages due to different genetic factors, with the latter being rare and unlikely to cause complete self-sterility in related species [20]. In *A. triloba*, uniform fruit drop occurs at 20 DAP, and similar cytological features, such as an undivided zygote and comparable endosperm development, are observed after self-pollination. Similar fruit drop has also been observed in other species with LSI, though the timing generally differs, occurring at 3–8 DDP in *Chorisia chodatii*, *C. speciosa*, *Tabebuia caraiba*, *T. ochracea* [70], *Dolichandra cynanchoides* and *Tabebuia nodosa* [45], *Spathodea campanulata* [71], and *Jacaranda racemosa* [72], and more rarely later, such as at 30 DAP in *Acca sellowiana* [73].

Few studies have explored post-fertilization events at the cellular level in species with LSI. However, cytological studies in LSI species consistently show a pattern in which endosperm development proceeds despite arrested embryo division [70, 73]. This pattern is considered a hallmark of postzygotic LSI, as it indicates that fertilization has occurred, but the incompatibility response is triggered after gamete fusion. In *A. triloba*, following CC pollination, the embryo remains quiescent until 18–19 DAP, when the first division typically occurs. Meanwhile, the endosperm begins dividing earlier and becomes multiseriate through longitudinal cell divisions [44]. In AI pollination, fertilization also occurs -as shown by the presence of two prominent nucleoli and large amorphous vacuoles, as reported in other angiosperms [74–77] - yet the embryo remains quiescent and fails to divide, while the endosperm continues dividing until it reaches 8–9 cells. These observations support the presence of an LSI mechanism in *A. triloba*, in which the incompatibility response acts post-fertilization and specifically affects embryo development.

Coordination between embryo and endosperm development has been studied to a limited extent, with most molecular and genetic mechanisms primarily evaluated in *Arabidopsis.* In *Arabidopsis*, the entry of the sperm is sufficient to trigger division of the central cell, although the presence of the parental genome is required for continued endosperm development [78]. Furthermore, an unidentified positive signal from the fertilized embryo has been reported to promote endosperm development [79]. In fact, the fertilized central cell enters mitosis immediately after fertilization, in contrast to the zygote, which remains quiescent for a short period [74]. Thus, in *Arabidopsis*, while he embryo remains relatively quiescent during the early stages of development, the endosperm is transcriptionally active [80]. A similar process may occur in *A. triloba*, where endosperm develops while the zygote remains in a quiescent stage, though for a longer duration compared to *Arabidopsis*.

Recent studies in *Arabidopsis* have reported that the central cell is arrested in the S phase by the RETINOBLASTOMA-RELATED 1 (RBR1) protein, which is later degraded following fertilization to allow progression beyond the S phase [81]. Interestingly, the RBR1 homolog in *A. triloba* is upregulated in CC pollination at 8 and 15 DAP, suggesting a regulatory role in compatible embryo-endosperm development.

### Transcriptomic divergences after self- and cross-pollination in *A. triloba*: insights into late-acting self-incompatibility

Beyond cellular-level differences, the first significant transcriptomic divergences between AI and CC pollination in *A. triloba* were observed at 8 DAP, revealing distinct biological pathways. In CC pollination, gene expression primarily promotes proliferation and development, whereas in AI pollination it is more associated with stress response and senescence. Key differences include processes typically activated after karyogamy in CC pollination, such as chromatin remodeling [82, 83], with genes like *SYP*, which appears to play a role in embryogenesis [84] and whose homolog in *A. triloba* was upregulated in CC pollination. Additionally, genes involved in molecular transport, potentially related to nuclear migration, were also differentially upregulated after CC pollination. Another notable difference at 8 DAP is the expression of embryo-defective genes. Nine of these genes were upregulated and three downregulated in CC pollination compared to AI pollination.

These broader transcriptomic trends are further supported by the expression of specific genes associated with early embryogenesis. For instance, among the DEG at 8 DAP, the one with the highest differential expression is an ankyrin-3-like gene. Ankyrins are thought to mediate protein-protein interactions, and one variant (ankyrin-6) has been reported to play a role in male and female recognition prior to fertilization in *Arabidopsis* [85]. Other genes involved in early embryogenesis, such as WOX8 and WOX9, which play a role in apical-basal axis formation [86, 87], are present as a single homolog in pawpaw, similar to what has been observed in *Annona cherimola* in our ongoing study (manuscript in review). This homolog was downregulated in *A. triloba* at 8 DAP in CC pollination compared to AI pollination. Another noteworthy gene, ZEU1, involved in early embryogenesis and that plays a role in DNA metabolism during the S phase of the cell cycle [88], was downregulated in CC crosses compared to AI crosses at 4 DAP, although it was not differentially expressed at 8 DAP. At 4 DAP, there were also some notable differences—although with low expression— such as a homolog of the AT4G21390 gene from the *S*-locus of the SSI incompatibility system, which is inactive in *Arabidopsis thaliana*, but not in *Arabidopsis lyrata* [89].

ARGONAUTE proteins, crucial components of the gene silencing pathway involved in developmental regulation, including embryogenesis, as seen with AGO10/ZLL [90, 91] and AGO5/9 [92] in *Arabidopsis*, were also differentially expressed. In our study, we observed an AGO-like protein (possibly AGO16) that was upregulated at 8 DAP and showed even greater differential upregulation at 15 DAP in compatible crosses. Additionally, homologs of AGO2/3, AGO4/5/6/7, and AGO8 were similarly upregulated at 15 DAP, with AGO6/8/9 showing the highest expression levels. Interestingly, the homolog of AGO1 was downregulated in compatible crosses.

Other genes implicated in embryogenesis, primarily studied in *Arabidopsis*, were also differentially expressed. Notably, homologs of DMP1/2, which are crucial for gamete fusion [93], were upregulated in AI crosses at 15 DAP. Particularly intriguing is the high expression and strong differential upregulation of the homolog of FIS3 in CC crosses at 15 DAP. FIS3 has a critical role in suppressing endosperm development until fertilization occurs [94]. However, at 15 DAP, it is important to consider that the developmental processes in CC and AI pollination differ significantly: while CC pollination involves pathways related to successful fruit development, AI pollination is more associated with plant organ senescence, likely related to imminent fruitlet drop. Thus, the genes involved in embryogenesis at 15 DAP should be interpreted within the context of these distinct biological processes.

Several candidate genes involved in post-zygotic LSI have been identified in other species. For example, in cacao, six classes of candidate genes were reported [26]. Among these, FAR1-like proteins and a serine/threonine-protein kinase were found to be upregulated in CC crosses in *A. triloba*. Specifically, four *FAR1*-like genes were upregulated at 4 DAP, three at 8 DAP, and a serine/threonine protein kinase was upregulated at 15 DAP. Conversely, eight FAR1-like genes and two serine/threonine protein kinases were downregulated in CC crosses compared to AI crosses in *A. triloba*.

In a separate study on cacao, 12 putative candidate genes were evaluated. Among them, orthologs of *BAM1*/*BAM2* were identified as putative candidates with higher expression levels in self-compatible crosses in cacao [23]. In *Arabidopsis*, BAM1, BAM2, and BAM3 are receptor-like kinases that play critical roles in plant development, particularly in meristem function and cell differentiation [95]. Interestingly, in our work, at 8 DAP, a *BAM3* homolog was upregulated in CC crosses compared to AI crosses and remained upregulated at 15 DAP, as did a *BAM1/2* homolog. Moreover, other candidates evaluated by Lanaud et al. [23] also showed higher expression in incompatible crosses at 15 DAP in *A. triloba*. These included homologs of a voltage-dependent L-type calcium channel subunit, a Zinc finger (AN1-like) family protein, and an ARM repeat-containing protein (*ARC1*).

## Conclusion

This study demonstrates that *Asimina triloba* employs both prezygotic protogynous dichogamy and postzygotic late-stage self-incompatibility (LSI) to prevent production of seeds from self-pollination, with LSI maintaining reproductive barriers despite prolonged stigmatic receptivity. Transcriptomic analysis shows distinct gene expression pathways between CC and AI pollination at 8 DAP, with minor differences detectable as early as 4 days, highlighting genes implicated in LSI and embryogenesis. These findings underscore conserved LSI mechanisms, aligning with studies in other species, and provide new insights into the evolution of self-incompatibility in early divergent angiosperms. By elucidating these processes in *A. triloba*, this study enhances our understanding of reproductive strategies in early-diverging lineages and lays the foundation for future research on the origins and diversification of self-incompatibility mechanisms in flowering plants.

## Supplementary Information

Supplementary Material 1: Table S1. Genes associated with embryo development in *Arabidopsis thaliana* and potential candidates for LSI identified in *Theobroma cacao*.

Supplementary Material 2: Table S2. Gene expression patterns based on RNA-seq analysis at 8 days after pollination (DAP).

Supplementary Material 3: Figure S1. Correlation analysis of transcriptomic profiles across developmental stages (4, 8, and 15 days after pollination) for both cross-compatible (CC) and auto-incompatible (AI) pollinations. Figure S2. Enriched GO Biological Processes of the specific genes in auto-incompatible (AI) samples at 15 DAP. Figure S3. Volcano plots (top) and Venn diagrams (bottom) of Differentially Expressed Genes (DEG) between cross-compatible (CC) and auto-incompatible (AI) pollinated plants. Figure S4. Network of the biological processes associated with differentially expressed genes (DEGs) between cross-compatible (CC) and auto-incompatible (AI) pollination at 8 days after pollination (DAP).

Supplementary Material 4: Figure S5. Heatmap of genes associated with embryo development in *Arabidopsis thaliana* and potential candidates for LSI identified in *Theobroma cacao* (Table S2).

## Data Availability

All relevant data can be found within the manuscript and its supporting materials. The sequencing data generated in this study have been deposited in the NCBI Sequence Read Archive (SRA) under BioProject ID PRJEB97068.
