# Supplementary material for: Late-acting self-incompatibility in Asimina triloba: implications for the evolution of self-incompatibility in angiosperms
Source: BMC Plant Biol. 2025 Dec 10;26:77. doi: 10.1186/s12870-025-07681-6 (PMC12802289; doi:10.1186/s12870-025-07681-6)
Supplement: Supplementary file 3 — Supplementary Material 3: Figure S1. Correlation analysis of transcriptomic profiles across developmental stages (4, 8, and 15 days after pollination) for both cross-compatible (CC) and auto-incompatible (AI) pollinations. Figure S2. Enriched GO Biological Processes of the specific genes in auto-incompatible (AI) samples at 15 DAP. Figure S3. Volcano plots (top) and Venn diagrams (bottom) of Differentially Expressed Genes (DEG) between cross-compatible (CC) and auto-incompatible (AI) pollinated plants. Figure S4. Network of the biological processes associated with differentially expressed genes (DEGs) between cross-compatible (CC) and auto-incompatible (AI) pollination at 8 days after pollination (DAP). [file 12870_2025_7681_MOESM3_ESM.pdf]

Late-Acting Self-Incompatibility in *Asimina triloba*: Implications for the Evolution of Self-Incompatibility in Angiosperms.

Cristina Ferrer-Blanco, Jorge Lora, Enrique Lopez-Gomez, Aynhoa Gomez-Olle, Noe Fernandez-Pozo, Jose I. Hormaza

Supplementary Material 3. Figures S1, S2, S3 and S4

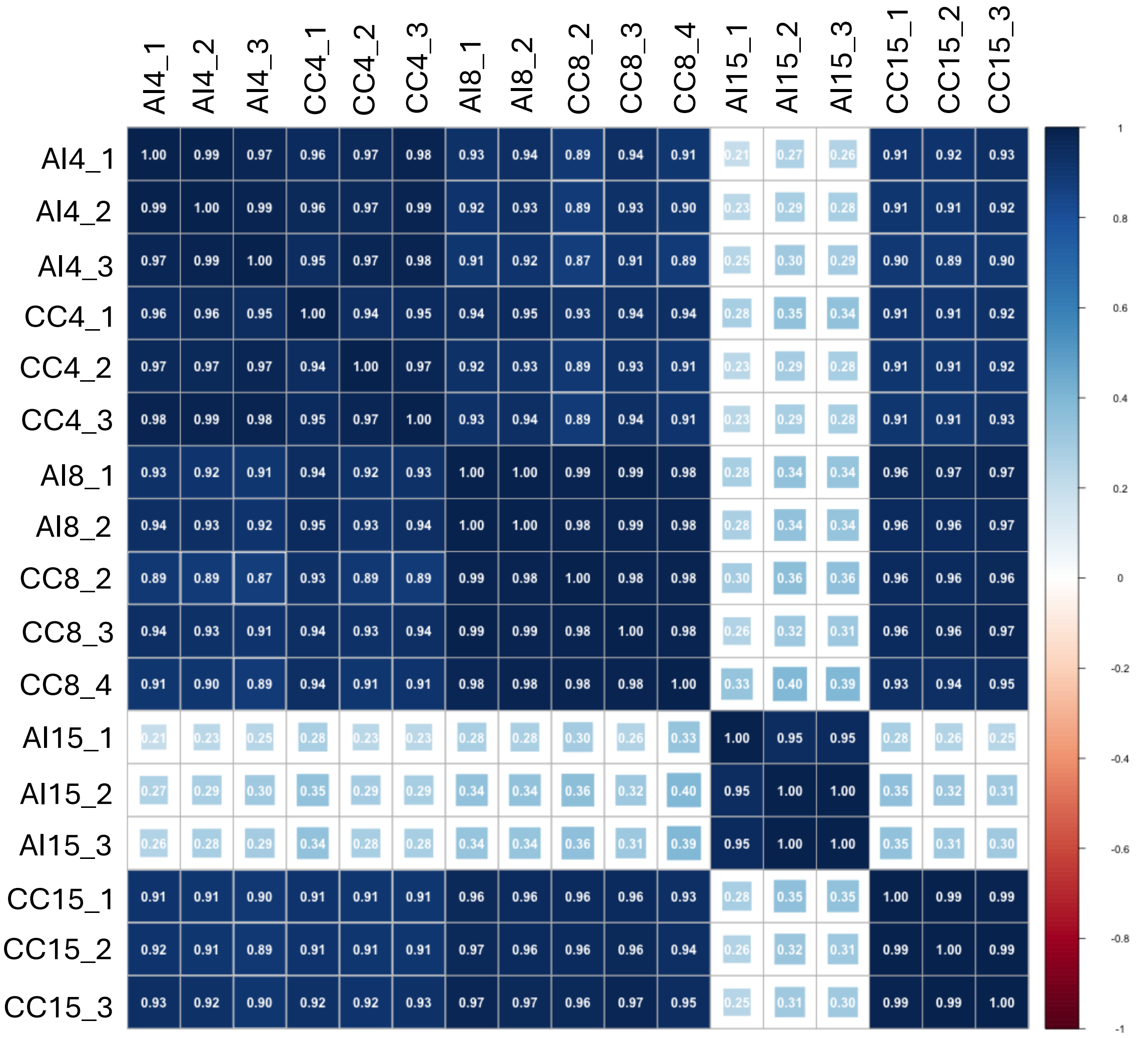

Fig. S1. Correlation analysis of transcriptomic profiles across developmental stages (4, 8, and 15 days after pollination) for both cross-compatible (CC) and auto-incompatible (AI) pollinations.

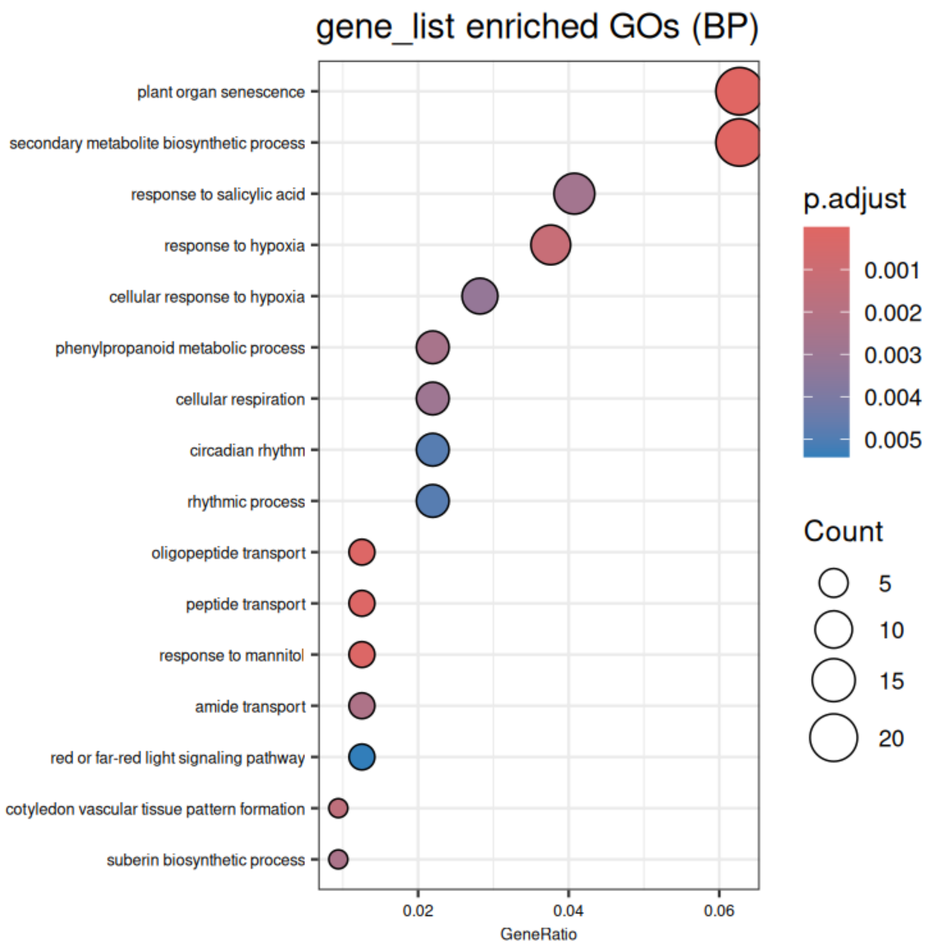

Fig. S2. Enriched GO Biological Processes of the specific genes in auto-incompatible (AI) pollination samples at 15 DAP.

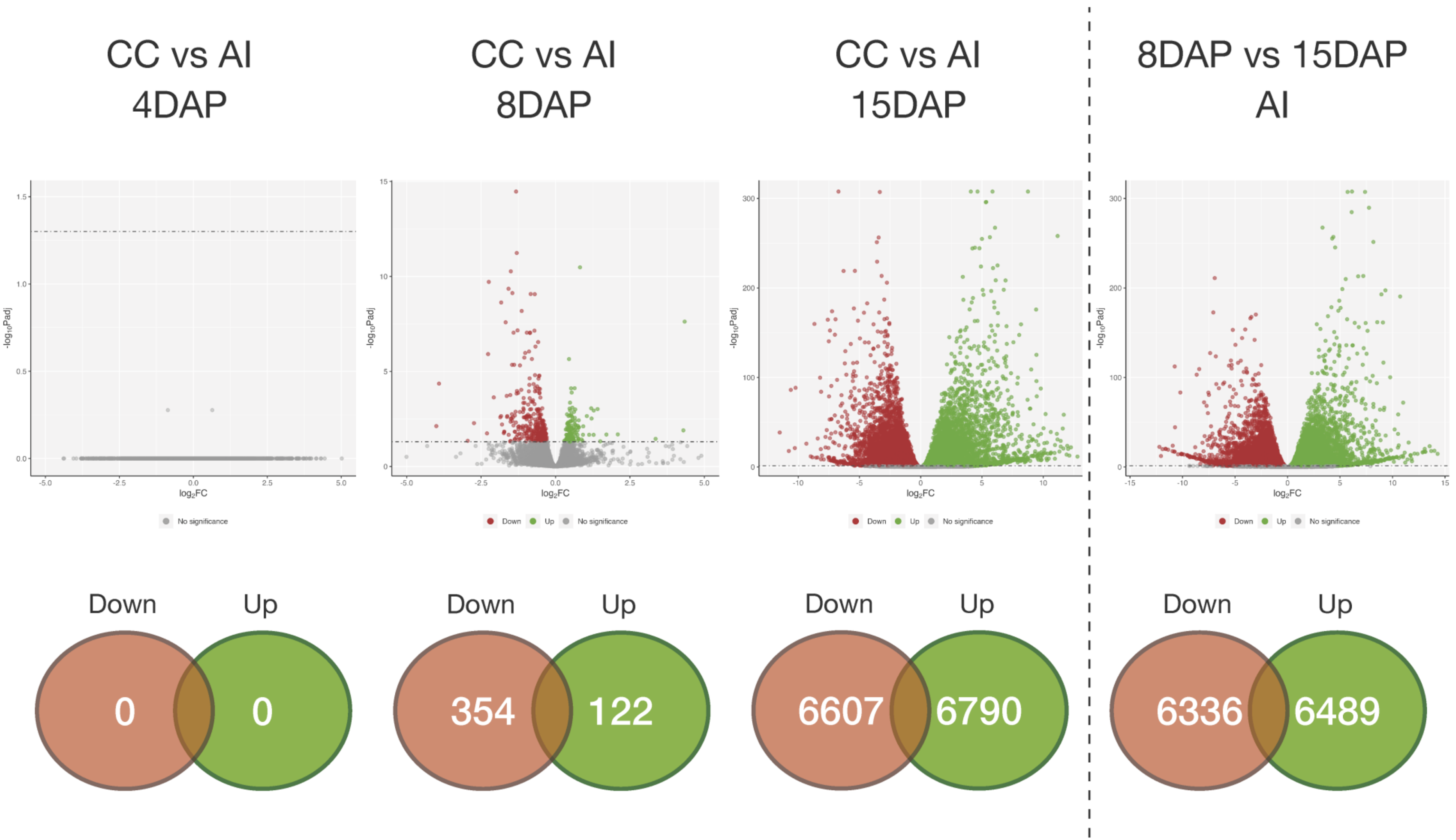

Fig. S3. Volcano plots (top) and Venn diagrams (bottom) of Differentially Expressed Genes (DEG) between cross-compatible (CC) and auto-incompatible (AI) pollinated plants at 4, 8 and 15 Days After Pollination (DAP) on the left of the dashed line, and between auto-incompatible plants at 8 and 15 DAP on the right. In comparisons between CC and AI (left), downregulated and upregulated genes of AI plants are shown in red and green respectively. In the comparison between 8 and 15 DAP in AI plants (right), downregulated and upregulated genes of AI plants at 15 DAP are shown in red and green respectively.

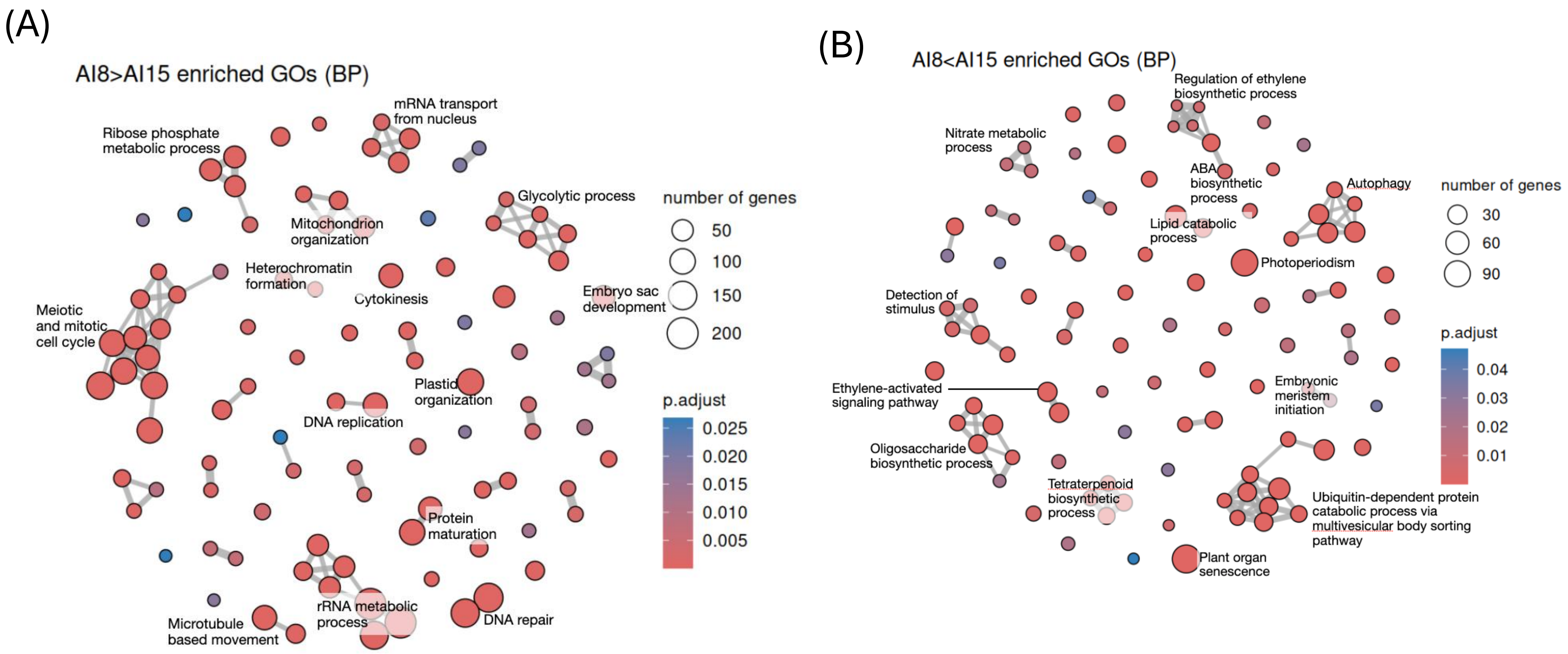

Fig. S4. Network of the biological processes associated with differentially expressed genes (DEGs) between cross-compatible (CC) and auto-incompatible (AI) pollination at 8 days after pollination (DAP). (A) Enriched processes in upregulated genes in CC pollination compared to AI pollination. (B) Enriched processes in upregulated genes in AI pollination compared to CC pollination.
