# Supplementary material for: Late-acting self-incompatibility in Asimina triloba: implications for the evolution of self-incompatibility in angiosperms
Source: BMC Plant Biol. 2025 Dec 10;26:77. doi: 10.1186/s12870-025-07681-6 (PMC12802289; doi:10.1186/s12870-025-07681-6)
Supplement: Supplementary file 4 — Supplementary Material 4: Figure S5. Heatmap of genes associated with embryo development in Arabidopsis thaliana and potential candidates for LSI identified in Theobroma cacao (Table S2). [file 12870_2025_7681_MOESM4_ESM.pdf]

Late-Acting Self-Incompatibility in *Asimina triloba*: Implications for Self-Incompatibility Evolution in Angiosperms.

Supplementary Material 4. Fig. S5. Heatmap of genes associated with embryo development in *Arabidopsis thaliana* (A-E) and potential candidates for LSI identified in *Theobroma cacao* (F) (Table S1).

(A) *S*-locus and hybrid incompatibility

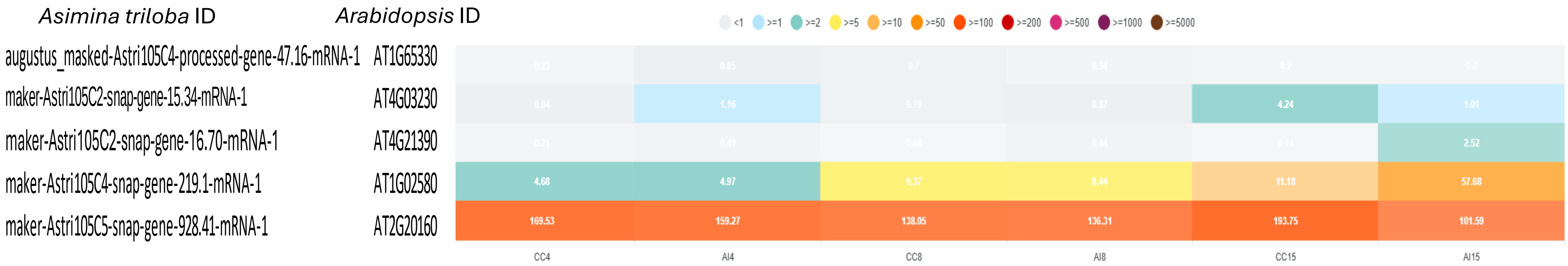

(B) Fertilization

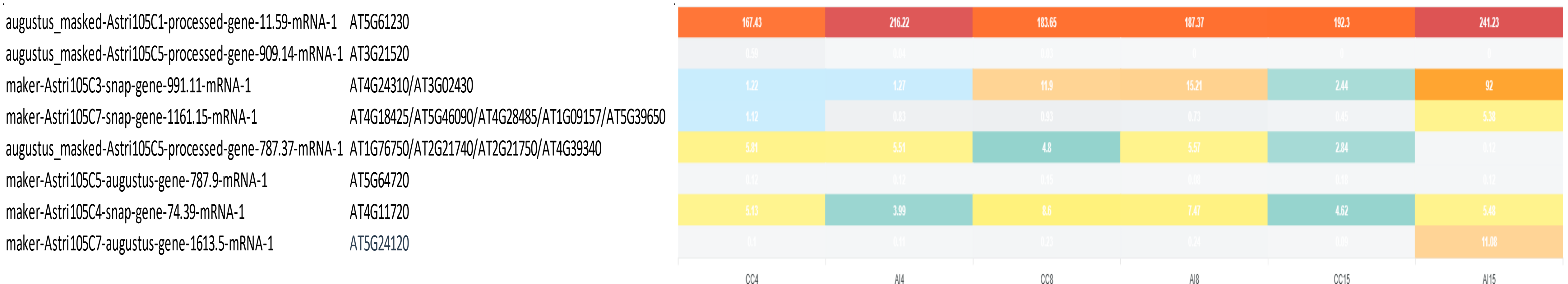

(C) Early embryogenesis

<1 · >=1 · >=2 · >=5 · >=10 · >=50 · >=100 · >=200 · >=500 · >=1000 · >=5000

		CC4	AI4	CC8	AI8	CC15	AI15
augustus_masked-Astri105C5-processed-gene-950.3-mRNA-	AT1G73360.1	104.01	99.05	75.29	71.21	56.91	8.84
maker-Astri105C1-augustus-gene-238.11-mRNA-1	AT5G59440	145.99	179.15	147.65	152.16	132.61	56.77
maker-Astri105C1-snap-gene-1006.24-mRNA-1	AT5G53040	8.27	11.21	3.93	3.97	4.06	0.08
maker-Astri105C1-snap-gene-297.16-mRNA-1	AT1G19850	5.99	5.74	5	4.63	3.62	0.51
maker-Astri105C2-snap-gene-1.20-mRNA-1	AT1G13980	126.17	111.45	133.04	124.77	152.94	165.58
maker-Astri105C2-snap-gene-358.28-mRNA-1	AT1G13980	0.01	0	0	0.01	0	0
maker-Astri105C2-snap-gene-358.29-mRNA-1	AT1G13980	0	0	0	0	0	0
maker-Astri105C2-snap-gene-358.31-mRNA-1	AT1G13980	0	0	0.01	0.01	0	0
maker-Astri105C3-augustus-gene-907.18-mRNA-1	AT1G23080	0.23	0.03	0.41	0.22	0.25	0
maker-Astri105C3-augustus-gene-953.52-mRNA-1	AT5G62270	49.7	55.61	44.61	47.83	45.39	10.53
maker-Astri105C3-augustus-gene-978.8-mRNA-1	AT2G01210.1	91.44	73.73	79.62	77.06	75	39.13
maker-Astri105C3-snap-gene-908.67-mRNA-1	AT1G23080	15.43	12.39	12.36	16.16	5.93	78.39
maker-Astri105C4-augustus-gene-897.4-mRNA-1	AT1G10717	0.08	0	0.07	0.03	0.04	0.97
maker-Astri105C4-snap-gene-278.38-mRNA-1	AT1G13980.1	0	0.05	0	0	0	0
maker-Astri105C4-snap-gene-296.23-mRNA-1	AT4G35230.1	16.76	15.95	19.11	18.59	14.72	29.32
maker-Astri105C5-augustus-gene-1029.5-mRNA-1	AT1G13800	66.1	59.27	64.93	59.81	51.91	67.17
maker-Astri105C5-augustus-gene-1065.6-mRNA-1	AT1G63700	21.77	21.69	26.08	25.58	23.49	20.87
maker-Astri105C5-augustus-gene-1070.52-mRNA-1	AT1G13980.1	37.39	38.08	24.12	20.08	32.9	26.35
maker-Astri105C5-augustus-gene-62.1-mRNA-1	AT2G38280	42.08	41.94	39.42	34.78	38.76	49.82
maker-Astri105C5-augustus-gene-884.6-mRNA-1	AT5G18580	68.8	60.55	71.56	63.59	68.46	90.75
maker-Astri105C5-snap-gene-3.13-mRNA-1	AT2G18510.1	286.04	275.61	318.61	336.16	263.33	147.44
maker-Astri105C6-snap-gene-179.10-mRNA-1	AT2G01210.1	25.06	23.22	26.58	27.98	23.49	82.31
maker-Astri105C6-snap-gene-274.48-mRNA-1	AT3G05870	67.17	53.69	71.43	63.46	110.72	26.71
maker-Astri105C6-snap-gene-63.25-mRNA-1	AT4G35230.1/AT2G17090	8.38	10.19	8.34	7.92	6.66	0.95
maker-Astri105C6-snap-gene-813.19-mRNA-1	AT1G73360.1	2.27	2.13	1.82	1.7	1.96	0.15
maker-Astri105C7-augustus-gene-1342.36-mRNA-1	AT5G59340	0	0	0	0.14	0.05	4.28
maker-Astri105C7-snap-gene-1112.11-mRNA-1	AT2G33880/ AT5G45980	102.33	125.84	94.03	79.92	84.33	0.49
maker-Astri105C7-snap-gene-151.3-mRNA-1	AT3G12280.1	149.96	151.41	122.3	98.37	109.18	58.17
maker-Astri105C7-snap-gene-717.12-mRNA-1	AT1G04550	24.22	22.48	20.59	18.73	22.62	5.81
maker-Astri105C7-snap-gene-95.2-mRNA-1	AT1G44900.1	36.1	28.11	17.61	16.36	11.81	58.59
maker-Astri105C7-snap-gene-970.37-mRNA-1	AT5G56270	0.12	0.24	0.15	0.27	0.03	0.01
maker-Astri105C8-augustus-gene-344.27-mRNA-1	AT1G13960.1	10.67	10.36	13.01	12.12	7.94	18.87
maker-Astri105C8-snap-gene-2.25-mRNA-1	AT5G56270	77.1	65.8	66.89	57.57	47.21	190.39
snap_masked-Astri105C1-processed-gene-879.14-mRNA-1	AT5G46570.1	39.49	37.14	43.39	45.82	31.89	36.24
snap_masked-Astri105C8-processed-gene-139.14-mRNA-1	AT2G18510.1	5.01	5.35	7.22	8.56	7.26	5.1

(D) Endosperm development

<1 >=1 >=2 >=5 >=10 >=50 >=100 >=200 >=500 >=1000 >=5000

		CC4	AI4	CC8	AI8	CC15	AI15
augustus_masked-Astri105C4-processed-gene-47.16-mRNA-1	AT5G48670	0.23	0.85	0.7	0.34	0.2	0.3
maker-Astri105C2-snap-gene-498.11-mRNA-1	AT3G20740	22.83	25.58	14.52	14.84	13.52	1.96
maker-Astri105C2-snap-gene-95.23-mRNA-1	AT5G58230	285.74	267.77	381.18	395.02	410.43	646.06
maker-Astri105C3-augustus-gene-1059.20-mRNA-1	AT2G35670	45.98	45.87	28.44	24.45	21.7	4.94
maker-Astri105C3-augustus-gene-927.0-mRNA-1	AT1G23320	39.35	44.24	41.45	39.63	40.83	49.26
maker-Astri105C3-snap-gene-862.1-mRNA-1	AT5G04560	33.15	31.64	34.87	32.71	38.56	23.39
maker-Astri105C4-augustus-gene-136.15-mRNA-1	AT1G48910	2.49	4.15	0.29	1.42	3.6	21.47
maker-Astri105C5-snap-gene-842.14-mRNA-1	AT4G02060	35.55	40.78	29.92	24.45	35.54	0.43
maker-Astri105C7-snap-gene-1254.44-mRNA-1	AT1G22015	47.19	46.38	37.52	39.14	37.26	63.3
maker-Astri105C8-augustus-gene-331.19-mRNA-1	AT5G60440	0	0	0	0	0	0

(E) Argonaute genes

		CC4	AI4	CC8	AI8	CC15	AI15
maker-Astri105C1-snap-gene-67.28-mRNA-1	AT5G43810	100.97	93.66	93.67	89.2	64.16	47.08
maker-Astri105C1-snap-gene-84.19-mRNA-1	AT5G21030	297.06	316.78	309.47	294.41	356.47	179.71
maker-Astri105C2-snap-gene-257.16-mRNA-1	AT1G31280/AT1G31290	13.89	12.69	7.97	7.78	11.51	4.08
maker-Astri105C4-snap-gene-183.23-mRNA-1	AT1G48410	731.48	767.69	1120.52	1006.83	944	2088.69
maker-Astri105C6-snap-gene-335.3-mRNA-1	AT2G27040/AT2G27880/AT2G32940/AT1G69440	244.99	228.73	295.83	276.92	258.84	120.17
maker-Astri105C7-augustus-gene-1362.32-mRNA-1	AT5G21150	24.23	26.51	24.37	24.52	21.69	6.97
maker-Astri105C8-snap-gene-90.48-mRNA-1		8.7	9.54	8.64	5.54	9.76	0.93

(F) Candidates LSI

<1 >=1 >=2 >=5 >=10 >=50 >=100 >=200 >=500 >=1000 >=5000

Gene	Arabidopsis homolog	CC4	AI4	CC8	AI8	CC15	AI15
augustus_masked-Astri105C1-processed-gene-160.61-mRNA-1	AT5G40190	7.2	6.8	8.09	8.44	7.62	16.96
augustus_masked-Astri105C4-processed-gene-150.63-mRNA-1	AT1G10240.1	75.35	78.92	102.1	100.46	93.16	90.27
maker-Astri105C1-augustus-gene-133.44-mRNA-1		16.27	13.84	23.06	18.4	17.51	20.13
maker-Astri105C1-snap-gene-171.24-mRNA-1		155.86	158.79	145.07	144.85	138.14	23.72
maker-Astri105C1-snap-gene-34.27-mRNA-1	AT1G51940.1	16.83	14.07	23.03	18.7	16.25	25.08
maker-Astri105C2-snap-gene-77.7-mRNA-1		76.03	59.42	80.8	66.77	76.08	148.81
maker-Astri105C3-augustus-gene-912.17-mRNA-1	AT5G50000	88.85	81.39	98.28	84.32	79.05	183.28
maker-Astri105C3-snap-gene-1074.39-mRNA-1	AT2G27110.1/AT4G38180.1	18.93	15.24	20.6	17.75	16.23	21.48
maker-Astri105C3-snap-gene-3.35-mRNA-1	AT4G38170.1	53.91	44.19	46.8	40.35	37.74	42.98
maker-Astri105C4-augustus-gene-227.35-mRNA-1	AT1G80010.1	11.84	9.75	10.67	9.93	9.7	15.76
maker-Astri105C5-snap-gene-850.24-mRNA-1	AT5G28530.1	73.96	76.52	67.99	60.49	60.25	109.36
maker-Astri105C6-augustus-gene-20.0-mRNA-1	AT5G65687	54.28	52.33	72.97	67.1	67.05	110.73
maker-Astri105C6-augustus-gene-21.1-mRNA-1	AT5G65700/AT3G49670	245.58	189.19	315.38	311.76	277.42	46.71
maker-Astri105C6-snap-gene-156.37-mRNA-1	AT3G28007.1	0.29	0.64	0.72	0.48	0.09	0.31
maker-Astri105C6-snap-gene-865.19-mRNA-1	AT5G55490	0.16	0.27	0.26	0.24	0.37	0.54
maker-Astri105C6-snap-gene-912.0-mRNA-1	AT5G16550	17.93	15.73	22.51	23.92	19.61	55.23
maker-Astri105C6-snap-gene-972.25-mRNA-1	AT3G17850.1	224.13	213.45	249.66	208.13	220.66	345.53
maker-Astri105C7-augustus-gene-1364.15-mRNA-1	AT3G11410.1	4.94	4.86	21.32	17.71	11.79	1.57
maker-Astri105C7-augustus-gene-999.1-mRNA-1	AT4G20270.1	181.93	157.24	159.93	176.38	134.21	1115.72
maker-Astri105C7-snap-gene-605.34-mRNA-1	AT1G52520.1 /AT4G19990.2 /AT2G32250.1	132.02	110.12	128.86	122.61	117.76	209.84
maker-Astri105C8-snap-gene-191.25-mRNA-1	AT3G49660	22.52	20.21	22.58	19.3	21.4	35.27
maker-Astri105C8-snap-gene-394.11-mRNA-1	AT3G28210	19.01	16.8	24.98	19.33	20.01	25.22
maker-Astri105C8-snap-gene-52.45-mRNA-1	AT4G38180.1	91.66	85.77	91.16	91.15	69.79	47.22
maker-Astri105C8-snap-gene-53.20-mRNA-1	AT4G38180.1	0.36	0.25	0.39	0.1	0.24	0.73
